# The severity of malnutrition in children with epidermolysis bullosa correlates with disease severity

**DOI:** 10.1038/s41598-021-96354-z

**Published:** 2021-08-19

**Authors:** Seema Manjunath, Rahul Mahajan, Dipankar De, Sanjeev Handa, Savita Attri, Banchha Nidhi Behera, Sadhna Lal Bhasin, Rishi Bolia

**Affiliations:** 1 Department of Dermatology, Venereology, and Leprology, Postgraduate Institute of Medical Education and Research, Sector 12, Chandigarh, India; 2 Department of Pediatric Biochemistry, Postgraduate Institute of Medical Education and Research, Sector 12, Chandigarh, India; 3 Department of Dietetics, Postgraduate Institute of Medical Education and Research, Sector 12, Chandigarh, India; 4 Department of Pediatric Gastroenterology, Postgraduate Institute of Medical Education and Research, Sector 12, Chandigarh, India

**Keywords:** Nutrition disorders, Skin diseases

## Abstract

WHO defines malnutrition as severe if the *z-scores* are less than − 3 Standard deviation (SD), moderate if between − 2 and − 3 SD and mild if between − 2 SD to – 1 SD. This study was aimed to assess nutritional aspects of Indian children suffering from EB and to evaluate the effect of severity of EB on the severity of malnutrition. In this study, pediatric EB patients were evaluated prospectively for baseline nutritional status using anthropometric parameters and WHO growth charts, and its correlation with disease severity using instrument for Scoring Clinical Outcomes for Research of Epidermolysis Bullosa-iscorEB. In second phase, an individualized diet chart was given to meet the energy, protein and micronutrients needs and its effects were observed after 6 months. The median age of participants was 3 years (IQR-9). Of 57 patients, malnutrition was seen in 40.35% patients (22.81%-moderate and 17.54%-severe), and significantly correlated with iscorEB (r = 0.45, p < 0.0001). On bivariate regression analysis, iscorEB was independently associated with moderate-to-severe malnutrition (p = 0.047; OR 1.038, CI 1.011–1.066). iscorEB enabled the identification of patients with moderate-to-severe malnutrition with an Area Under Receiver Operating Curve (AUROC) of 0.72 (95%CI 0.58–0.85; p < 0.005). In phase 2, there was significant improvement in nutritional status in children with recessive dystrophic EB (RDEB) and dominant dystrophic EB (DDEB) subtype (p < 0.0001). The severity of malnutrition in EB children significantly correlates with disease severity, and is an independent predictor of moderate-to-severe malnutrition.

## Introduction

Over the years, the definition of pediatric malnutrition has undergone conceptual change with an emphasis on etiology-related definitions and etiology-related interventions, and the role of *z* scores in clinical practice. According to WHO, malnutrition refers to imbalance between the supply of protein and energy and body’s demand for them to ensure optimal growth and function. WHO categorizes malnutrition as severe if the *z-score* (weight for height and height for age) are less than – 3 Standard deviation (SD), moderate if *z-score* (weight for height and height for age) are between – 2 and – 3 SD, mild malnutrition if values are between – 2 SD to – 1SD^1^. The definitions for malnutrition were revised in the year 2006 by WHO to include six categories for undernutrition—stunted and severely stunted, underweight and severely underweight, and wasted and severely wasted^2^. The nutritional needs are increased for patients with epidermolysis bullosa (EB) due to chronic inflammation, recurrent secondary infection, and systemic involvement. Reduced food intake and enhanced nutritional needs together contribute to malnutrition in EB patients, leading to interruption in growth, puberty and wound healing. Hence, many children severely affected with EB have a varying degree of retardation of growth with the majority of them unable to meet the 5th percentile curves for weight/height^3^. In EB, children with minimal blisters and no gastrointestinal system involvement, the nutritional needs are comparable to those of age and sex-matched healthy children^4^. Since there is a lack of data regarding the frequency of malnutrition among children with EB from the developing world, this study was aimed to assess nutritional aspects of Indian children suffering from EB and to evaluate the effect of severity of EB on the severity of malnutrition.

## Materials and methods

This was a single center, prospective longitudinal study conducted over a period of 18 months (July 2017 to December 2018) at the Department of Dermatology, Venereology and Leprology, PGIMER, Chandigarh**.** The study was started and conducted after approval of the study protocol by the Institute’s Ethics Committee according to the guidelines set up by ICMR (1994) and Helsinki declaration (modified 2000). After obtaining written informed assent/consent from the children/parents, patients (aged less than 18 years) with the clinical diagnosis of EB were grouped into major subtypes—epidermolysis bullosa simplex (EBS), junctional epidermolysis bullosa (JEB), dystrophic EB (DEB) including both recessive dystrophic EB (RDEB), dominant dystrophic EB (DDEB), and Kindler syndrome (KS)—according to Clinical Diagnostic Matrix (CDM) and confirmation by transmission electron microscopy and/or immunofluorescence antigen mapping^5^.

### Phase I: Anthropometry and disease severity

A detailed history followed by a complete physical examination was recorded in case record proforma. The severity of EB was calculated using ‘iscorEB’, including clinician and patient score^6^. Besides, physical examination was focused on the anthropometric examination like weight, height, head circumference, mid-arm circumference, and weight for age, height for age, weight for height and body mass index (BMI) and comparison with the age-appropriate World Health Organization (WHO) growth charts to assess whether the child had any features of malnutrition^7^. They were categorized into having severe malnutrition if the *z-score* (weight for height and height for age) are less than – 3 Standard deviation (SD), moderate if *z-score* are between – 2 and – 3 SD, mild if values are between – 2 SD to – 1SD^1^. Table 1 enlists the laboratory investigations done in patients.

**Table 1:**
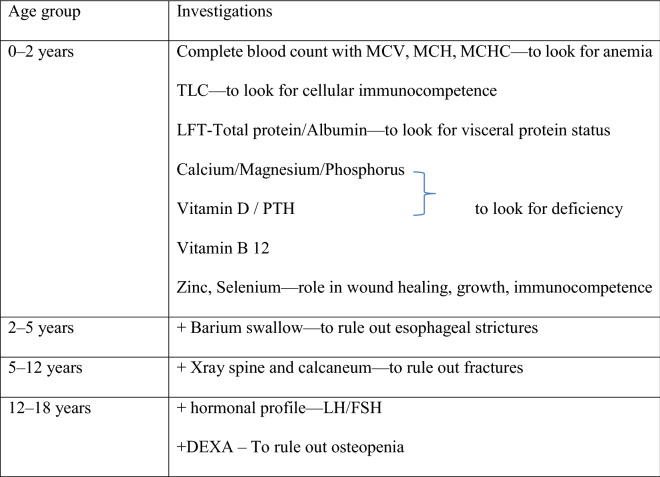
List of laboratory investigations undertaken in the study cohort.

*MCV* Mean corpuscular volume, *MCH* Mean corpuscular hemoglobin, *MCHC* Mean corpuscular hemoglobin concentration, *TLC* Total leucocyte count, *LFT* Liver function test, *PTH* Parathyroid hormone, *LH* Luteinizing hormone, *FSH* Follicle-stimulating hormone, *DEXA* Dual-energy X-ray absorptiometry.

### Phase II: Nutritional advice

The objective of the nutritional advice for children with Epidermolysis Bullosa (EB) was to provide more than adequate macronutrients as well as micronutrients, minimize the nutritional deficiencies, improve bowel functions, enhance immunological status, optimize wound healing, promote proper body growth as well as pubertal and sexual development and also is to minimize the stress caused by prolonged feeding time.

Prior to nutritional advice given to parents of an EB child, a detailed history was documented in a precise format with special emphasis on dietary intake with the help of 24-h recall method. Dietary history included typical meal pattern, consistency of food, frequency of meals, time taken over average meals, nutrient supplements if any, reason for difficulty in food intake according to child/ caretaker. Nutrient value of dietary intake and requirements were assessed as per guidelines by National Institute of Nutrition, Hyderabad^8^. After this, an individualized diet chart was given to meet the energy, protein and other nutrients needs of the child with EB and also more emphasis were given for frequent follow ups to monitor the improvement of the child (supplemental Fig. 1). Children with EB often requires more calories than usual. Thus, their diet plan was made by providing 100% to 150% of the Estimated Energy Requirement (EER) which is part of Dietary Reference Intake (DRI). If growth was not satisfactory based on follow up assessment, a gradual increase in energy was initiated. Due to excess protein loss through blisters as well as recurrent infections, the protein needs of the child with EB are greater than those healthy peers of matching gender and age. To maintain positive nitrogen balance, diet plans were calculated by providing 115–200% of the estimated average requirement as per Recommended Dietary Allowance (RDA)^9^.

**Figure 1:**
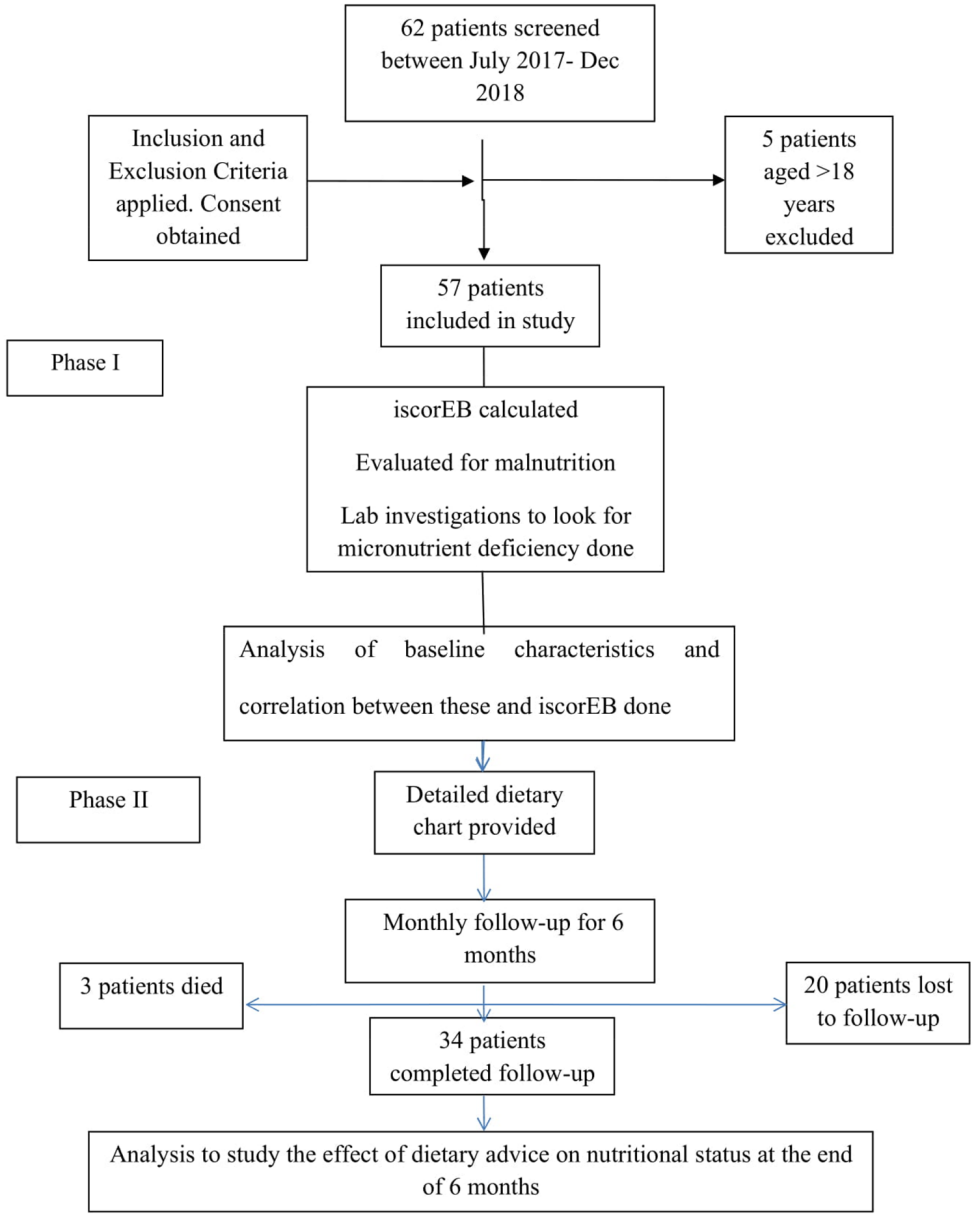
Flow Diagram of the study.

Adequate amounts of carbohydrate, especially complex carbohydrates were included in their daily diet plan to overcome the problem of constipation. Also, adequate amounts of fats such as butter/ghee if needed were provided as per RDA in their menu to increase the calorie need and palatability. While planning menu foods rich in vitamins like A, C, D, B6, B12, thiamine, riboflavin and minerals like Zinc, calcium and iron were well taken care in their daily diet.

At the end of the diet counseling, parents of child with EB were imparted the knowledge of high energy giving foods and how to prepare delicious and nutritious recipes in different consistency (thick liquid, semisolid, pureed) to improve their child’s taste and intake and given recipe handouts written in Hindi/English/Punjabi.

Since frequency, quantity and consistency of meals/feeds are very important factors while giving nutritional advice to children with EB, parents were advised to give small frequent meals/feeds customized to local tastes. In each follow up visit, patients with EB were assessed for the present intake and calculated as per the ICMR food values and then advised modified diets/feeds as per the child’s need.

Based on the 2010 recommendations of the Indian Council of Medical Research^10^, daily supplementation of a common commercially available multivitamin syrup was also supplemented. Micronutrient deficiency detected in laboratory evaluation was treated as per pediatrician advice. Esophageal dilation was done in patients with esophageal strictures detected on barium swallow by a pediatric gastroenterologist. Patients were followed up every month for at least 6 months. Repeat anthropometry and laboratory investigations were done at 6 months.

### Statistical analysis

The normality of quantitative data was checked by measures of Kolmogorov Smirnov tests of normality. Normally distributed data were compared using one-way ANOVA followed by post hoc multiple comparisons test. For the skewed data such as the baseline clinical and anthropometric variables, the Kruskal–Wallis test followed by the Mann–Whitney test was applied (Table 2). For comparison of time-related variables of skewed data such as the serial change in anthropometric characteristics, the Wilcoxon signed-rank test was carried out (Table 1S). Spearman correlation coefficients were calculated to see the relationship between different variables. To find an independent predictor for moderate to severe malnutrition, logistic regression analysis was carried out. Area Under Receiver Operating Characteristic (AUROC) curve was calculated to find maximal cut-off values of iscorEB to detect malnutrition. All statistical tests were two-sided and performed at a significance level of α = 0.05. The analysis was conducted using IBM SPSS STATISTICS (version 22.0, SPSS, Chicago, Illinois, USA).

**Table 2:** Baseline clinical characteristics in study subgroups.

	Study cohort	EBS	JEB	RDEB	DDEB	P value (Kruskal–Wallis H test)
Total number of patients	55	13	8	26	8	
The median age in years (IQR)	5 (9.40)	3 (10)	0.04 (7)	2 (8)	7 (11)	0.27
Gender	Female (%)	24 (42.10)	3 (23.10)	3 (37.50)	15 (57.70)	0.23
	Male (%)	33 (56.90)	10 (76.90)	5 (62.50)	11 (42.30)	
Gastrointestinal system involvement (%)	35 (61.40)	3 (23.10)	8 (100)	22 (84.60)	2 (25)	**0.001**
Dental involvement (%)	21 (47.70)	1 (9.10)	2 (66.70)	15 (75)	3 (37.50)	**0.019**
Mean iscorEB	36.02 ± 23.67	20.73 ± 13.65	50.58 ± 26.09	44.89 ± 21.86	25.86 ± 19.17	**0.012**

Baseline anthropometry parameters in each clinical category						
		EBS; n = 13	JEB; n = 8	RDEB; n = 26	DDEB; n = 8	P-value (Kruskal–Wallis H test)
Median weight (IQR) (kg)		15.00 (27)	3.00 (12)	8.50 (15)	16.00 (35)	0.04
Median weight for age (*z-score)* (IQR)		− 1.0 (1)	− 2.0 (2)	− 2.0 (2)	− 2.5 (2)	0.63
Median height (cms) (IQR)		95.00 (59)	49.50 (88)	80.00 (60)	109.50 (68)	0.10
Median height For age (IQR) (*z-score)*		− 1 (1)	− 1 (2)	− 2 (2)	− 2 (3)	0.47
Median Weight for height (*z-score)* (IQR)		− 1.0 (2)	− 3.0 (3)	− 1.0 (2)	− 1.0 (1)	0.48
Median head circumference (cms) (IQR)		50.00 (18)	34.75 (5)	41.75 (14)	49.50 (8)	0.07
Median head circumference for age (*z-score)* (IQR)		0	0.5 (1)	0 (2)	0 (2)	0.62
Median mid-upper arm circumference (cms) (IQR)		13.50 (1.60)	12.00	12.00 (2)	13.90 (3.10)	0.40
Median mid-upper arm circumference for age (*z-score)* (IQR)		1.0 (1)	–	1.0 (1)	1.0 (2)	0.59
Median BMI (kg/m^2^)(IQR)	14.90 (6.30)	13.70	13.40 (7.40)	14.75 (6.00)	19.20	0.09
Median BMI for age (*z-score)* (IQR)	1.0 (1)	–	1.0 (1)	1.0 (2)	–	0.67

*EBS* Epidermolysis bullosa simplex, *JEB* Junctional Epidermolysisbullosa, *RDEB* Recessive dystrophic Epidermolysisbullosa, *DDEB* Dominant dystrophic Epidermolysisbullosa, *KS* Kindler syndrome, *IQR* Interquartile range.

### Ethics approval

PGIMER Chandigarh Institute’s Ethics Committee NK/4227/MD/1544-45. The study was started and conducted after approval of the study protocol by the Institute’s Ethics Committee according to the guidelines set up by ICMR (1994) and Helsinki declaration (modified 2000).

## Results

Sixty-two patients with EB were screened and 57 eligible patients were included in the study as shown in the flow chart (Fig. 1). Of the 57 patients, 34 had dystrophic EB [26 (45.61%) patients had RDEB (16 patients—RDEB generalized severe type, 10—RDEB generalized intermediate type), and 8 (14.04%) patients had DDEB],13 (22.81%) patients had EBS (8 patients had localized type EBS, 3 patients had generalized intermediate type EBS, 2 had generalized severe type EBS), 8 (14.04%) patients had JEB (3 patients—JEB generalized intermediate and 5 patients—generalized severe type of JEB) and 2 (3.51%) patients had Kindler syndrome.

### Baseline clinical characteristics of the study cohort

Table 2 summarizes the baseline clinical data and the anthropometric characteristics of each subcategory. Of 57 patients, 35 (61.4%) patients had gastrointestinal system involvement. Oral mucosal erosions were present in 3 (8.6%) EBS, 8 (22.9%) JEB, 22 (62.9%) RDEB, 2 (5.7%) DDEB patients. Dysphagia was reported by 2 (5.7%) JEB patients and 12 (34.2%) RDEB patients. Ankyloglossia was seen in 10 (28.5%) RDEB patients. Constipation was reported by 3 (8.6%) EBS patients, 5 (14.2%) JEB patients, 18 (51.4%) RDEB patients and 2 (5.7%) DDEB patients. Perianal lesions were seen in 4 (11.4%) JEB and 8 (22.8%) RDEB patients. Microstomia was observed in 1 (2.8%) JEB and 9 (25.7%) RDEB patients. Gastroesophageal reflux symptoms were reported by 2 (5.7%) JEB and 2 (5.7%) RDEB patients. Thirteen patients aged < 6 months lacked dentition and hence were advised oral hygiene and care and were kept on follow up to be evaluated later. Of remaining 44 patients, 21 patients (47.73%) had dental involvement in the form of dental caries (13, 61.9%), enamel loss (2, 9.5%), crowding of teeth (10, 47.6%) and early loss of tooth (3, 14.2%).

**Figure 2:**
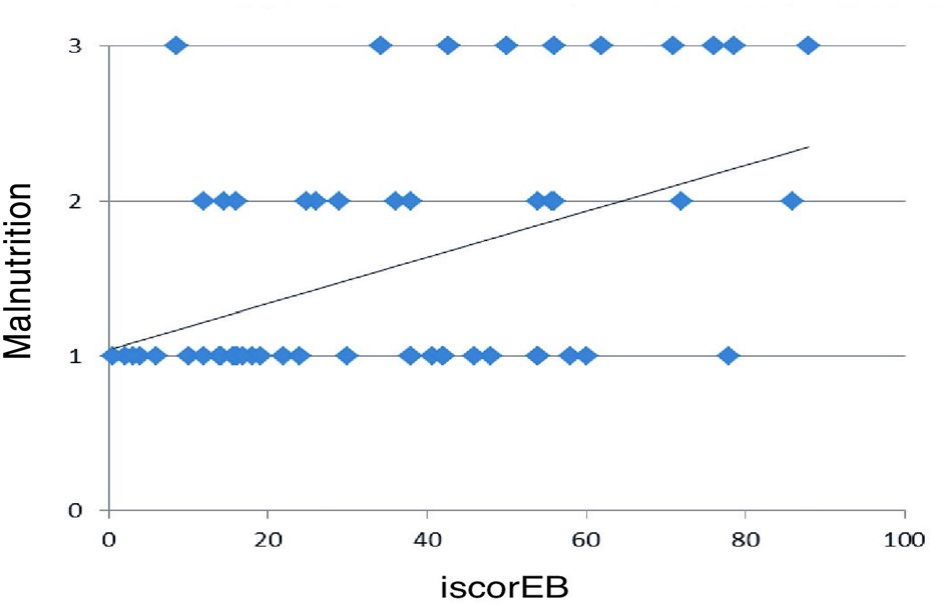
Scatter plot showing the correlation between iscorEB and malnutrition. (Y axis—3-severe malnutrition, 2-moderate malnutrition, 1-mild/ no malnutrition).

Thirty-four (59.6%) patients had normal/mild malnutrition, 13 (22.8%) patients had moderate and 10 (17.5%) patients had severe malnutrition. Out of 30 children aged below 5 years, 51.8% were underweight (low weight for age), 40% were stunted (low height for age) and 43.47% were wasted (low weight for height). There was no significant difference in the frequency of mild/normal, moderate and severe malnutrition among the various subtypes of EB (p = 0.29; chi square test). Severe malnutrition was most commonly seen in 9 dystrophic EB patients—7 RDEB (30.4%), 2 DDEB (8.7%), and 1 JEB (4.3%) patient. A moderate degree of malnutrition was seen in 6 RDEB (26.08%), 3 DDEB (13.04%), 2 JEB (8.7%) and 2 EBS (8.7%) patients. Higher iscorEB (56.72 ± 23.85) scores were seen in those with severe malnutrition compared to those with moderate malnutrition (40.00 ± 23.12) and normal/mild malnutrition (28.41 ± 20.14; p = 0.002), There was a statistically significant correlation between malnutrition and iscorEB with a moderate strength (Pearson coefficient r = 0.45, p < 0.0001; Fig. 2).

**Figure 3:**
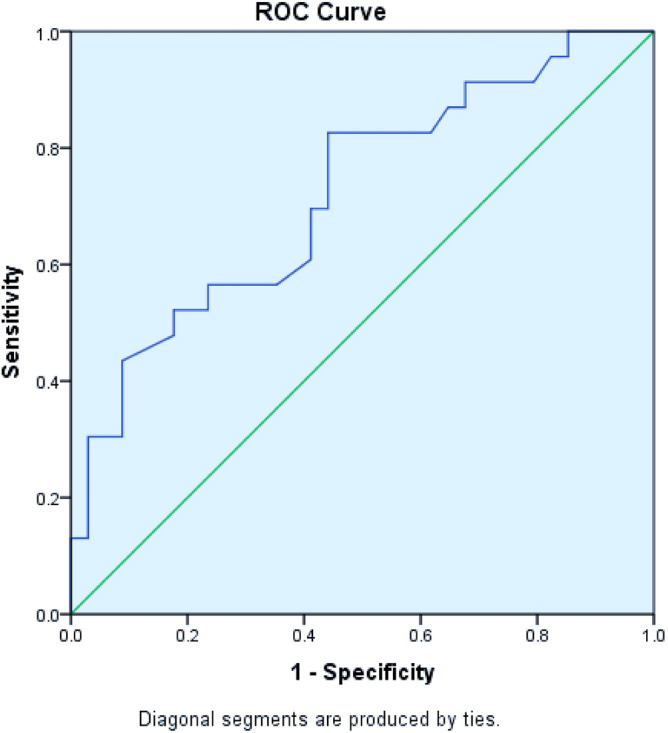
AUROC of the iscorEB when used to predict moderate to severe malnutrition.

We tried to find out factors associated with moderate to severe malnutrition. On bivariate analysis, it was significantly associated with gastrointestinal system involvement (p = 0.032) and iscorEB (p = 0.005) but not with age (p = 0.15), gender (p = 0.71), disease subtype (p = 0.12), and dental involvement (p = 0.07). On bivariate regression analysis, only iscorEB was independently associated with moderate to severe malnutrition (p = 0.047; OR = 1.038, CI = 1.011–1.066). iscorEB enabled the identification of patients with moderate to severe malnutrition with an AUROC of 0.72 (95% CI 0.58–0.85; p < 0.005, Fig. 3). At an estimated cut-off value of 24.5, iscorEB exhibited a sensitivity and specificity of 82.6% and 55.9%. Using the logistic regression model, an iscorEB value of more than 24.5 was associated with a markedly increased risk of moderate to severe malnutrition (p = 0.05; OR = 5.12, 95% CI = 1.00–26.15). Forty-one out of 57 patients underwent laboratory evaluation (Table 3). Eight patients had mild anemia (10–11 g/dl), 18 patients had moderate anemia (7–10 g/dl) and 1 patient had severe anemia (< 7 g/dl). Vitamin B12 deficiency was observed in 20 patients. Mean iscorEB was higher in those with moderate anemia (48.71 ± 24.20) and severe anemia (50.00) compared to those with mild anemia (23.36 ± 20.28) and normal hemoglobin (29.12 ± 21.65; p < 0.003). Vitamin D levels were found to be in sufficient in 22 patients, deficient in 12 patients. Low serum zinc and selenium levels were seen in 4 patients each. Among other laboratory parameters, total leucocyte count (p = 0.005; Kruskal Wallis test) and red blood cell distribution width (p = 0.039; Kruskal Wallis test) was significantly different among the disease subtypes.

**Table 3:** Laboratory findings of patients in the study cohort.

Parameters	Subgroups	Number of patients (%)
Hemoglobin (gm/dl)	< 7	1 (2.4)
	7–10	18 (43.9)
	10–11	8 (19.5)
RBC count (million/µl)	< 3	21 (51.2)
	> 4.5	2 (4.9)
MCV (fl)	< 74	22 (53.66)
	> 108	2 (4.88)
MCH (pg)	< 25	16 (39)
	> 35	1 (2.44)
MCHC (g/dl)	< 30	14 (34.15)
	> 36	1 (2.44)
Vitamin B12 (pg/ml)	< 211	20 (49)
Vitamin D (ng/ml)	< 10	12 (29.3)
	10–30	22 (53.6)
PTH (pg/ml)	< 15	1 (2.44)
	> 65	9 (21.95)
Total protein (g/dl)	< 6.4	18 (44)
Serum albumin (g/dl)	< 3.4	14 (34)
Calcium	< 8.8	18 (43.9)
	> 2.55	20 (48.8)
Phosphorus	< 2.7	2 (4.9)
Zinc	Below Normal range for age	4 (9.8)
Selenium	Below Normal range for age	4 (9.8)

Five of the 14 patients who underwent barium swallow showed upper esophageal strictures and underwent esophageal dilation (number of sessions ranged between 15 and 20). Of the 57 patients, 34 who followed dietary advice as advised by a dietician experienced a significant increase in mean weight and height (P < 0.0001). Mean follow-up duration was 6 months. Mean weight increased from 15.53 ± 15.02 kg to 17.87 ± 14.71 kg, and mean height increased from 92.64 ± 36.94 cm to 97.55 ± 34.14 cm. Similarly, baseline weight for age increased significantly from − 1.74 ± 1.13 to − 1.33 ± 1.10 at the end of 6 months with a corresponding increase in Z-scores (P = 0.01). Height for age increased from − 1.47 ± 0.98 to − 1.28 ± 1.11; and weight for height increased from − 1.43 ± 1.19 to − 1.04 ± 0.97 but improvements in these parameters were statistically non-significant (P = 0.11 and P = 0.10 respectively; Wilcoxan-signed rank test) (Table 1S). Among these 34 patients, the frequency of severe malnutrition decreased significantly from 26.4% to 8.8%. Improvement in nutritional status was statistically significant in the overall study cohort particularly due to significant improvement in RDEB (p < 0.0001) and DDEB (p < 0.009) subtypes (supplementary table 1).

## Discussion

The nutrition in the first few years of the life of a child is crucial concerning the overall growth and development and any factor that interferes with adequate nutritional intake during this period predisposes to significant failure to thrive. In the present study, malnutrition was seen in 40.35% patients, and significantly correlated with severity of disease. In fact, the disease severity was an independent determinant of severity of malnutrition. Also, there was significant improvement in nutritional status in children with dystrophic EB (RDEB) and dominant dystrophic EB (DDEB) with dietary advice. In our study, the median age at presentation of 3 years though comparable to other Indian studies^11^, reflects the delay in presentation to a tertiary care institute with specialized EB services in a developing country due to poor access to health care, and a lack of information and knowledge about the disease among parents and primary care physicians. There was a male predominance in the present study (M:F = 1.3:1) comparable to another Indian study by Hiremagalore et al.^12,13^.

Pediatric malnutrition can be illness-related and non-illness-related. Since there was no control group in the present study, we compared our data with the data in the National Family Health Survey 4(NFHS 4)^13^, which showed that 35.7% of Indian children below 5 years were underweight, 38.4% were stunted, 21% were wasted. Compared to children in NFHS 4 survey, a higher frequency of EB children was underweight and wasted with a slightly higher frequency of stunting. This can be attributed to a wide range of gastrointestinal complications affecting EB children in 69.2% of patients with moderate malnutrition and 90% of patients with severe malnutrition. Dental involvement was seen in a much higher proportion of patients with moderate to severe malnutrition although it did not achieve statistical significance. In EB patients, reduced food intake and enhanced nutritional needs together contribute to significantly enhanced risk of malnutrition, leading to interruption in growth, delayed puberty and wound healing^3,9^. Hence, the management of such comorbidities can help improve the nutritional status of EB children. Severe malnutrition was seen predominantly in females (M:F ratio = 2:3). This female predominance in malnutrition is multifactorial and is also reported in NFHS survey^14^.

Severe malnutrition was most commonly seen in RDEB (26.9%), followed by DDEB (25%), and JEB (12.5%). However, the disease subtype was not found to have a significant bearing on the frequency of malnutrition (p = 0.10). Few previous studies on this aspect have also reached similar conclusions with the frequency of malnutrition and failure to thrive being higher in patients with recessive dystrophic EB and junctional. Birge et al. found that out of 80 EB children, only 22% with EBS were having malnutrition whereas 77% with dystrophic EB and 57% with junctional EB had malnutrition. Fine et al. reported that 39% of RDEB, 2% of DDEB, 78% of children with JEB Herlitz type and 43% of those with non-Herlitz type were found to have malnutrition^15–17^. On correlating malnutrition with iscorEB, there was a statistically significant correlation with a moderate strength of association. Additionally, iscorEB was found to have an independent association with malnutrition (p = 0.005; OR = 1.038, 95%CI = 1.011–1.066) with a much stronger association at a cut-off level of 24.5. Since the disease severity goes hand in hand with malnutrition risk, it also alerts the treating physician about the need for an urgent nutritionist or pediatric gastroenterologist consultation.

The mean iscorEB was found to be higher in those with moderate and severe anemia compared to those with mild anemia. According to the Australasian EB registry^18^, 25.8% of pediatric EB patients were anemic and mostly seen in RDEB and JEB generalized severe type. Nearly 30% of patients had vitamin D deficiency. Mean iscorEB was higher in those with deficiency followed by those with insufficient levels (45.12 ± 27.61) compared to those with sufficient vitamin D levels (26.20 ± 22.97) but was not statistically significant (P < 0.259). Hypocalcemia could be due to decreased dietary intake or decreased absorption and thus affecting bone mineralization. The cause for hypermagnesemia could not be identified and patients did not manifest any symptoms. These observations are similar to other studies^19–23^.

To ensure supplementation of required calories, proteins and fibers, consistency, frequency, type of food were individualized based on age, severity of EB, severity of dysphagia. The total amount of calories was slowly built up by gradually increasing every week to reach the required level. This helped to overcome difficulties in dietary intake in children. For those with dysphagia, esophageal dilation was done which helped to increase the dietary intake to required level.

After following dietary advice for 6 months, there was a significant improvement in weight and height compared to baseline. After following dietary advice for 6 months, 29.4% had malnutrition (20.58%-moderate, 8.82%-severe; and 70.58% patients had normal/mild malnutrition (p < 0.0001). A study by Zhang et al. in infants found improvement in weight for age, height for age and weight for height scores after following dietary advice for 9–18 months when compared with the control group^24^. Since our follow up duration was only 6 months, an extended follow-up period might show even greater improvement in stunting and other anthropometric parameters. The improvement in nutrition especially in those with malnutrition by offering 100% to 150% of the EER and 115–200% of the estimated average requirement of protein underscores the increased demands to be met in EB children compared to their normal counterparts. Improvement in the frequency of malnutrition following dietary advice highlights the importance of diet in the management of overall growth and severity of disease in EB children. Better dietary intake helps to counter the detrimental effects of disease complications and increased demands because of the chronic inflammatory state. The barriers towards attaining normal nutritional status in 29.4% of patients in spite of dietary management could be the complications associated with the disease, inadequate parental education, and poor socioeconomic status and lack of initiative and encouragement at home for the same.

Despite our best efforts, our study had a few limitations. The results should be validated in a much larger group in the developing world preferably in a multicentric setting. Moreover, the effect of dietary advice was not compared with the control group as the children normally tend to gain weight and height during this period of growth and development.

## Conclusions

Moderate to severe malnutrition is common in children with EB and significantly correlates with disease severity as assessed by iscorEB. IscorEB was found to be independently associated with moderate to severe malnutrition. There was significant improvement in nutritional status in overall study cohort, especially in children within RDEB and DDEB subtype after dietary change.

## Supplementary Information

Supplementary Information.
