# Supplementary material for: The severity of malnutrition in children with epidermolysis bullosa correlates with disease severity
Source: Sci Rep. 2021 Aug 19;11:16827. doi: 10.1038/s41598-021-96354-z (PMC8377149; doi:10.1038/s41598-021-96354-z)
Supplement: Supplementary file 1 — Supplementary Information. [file 41598_2021_96354_MOESM1_ESM.docx]

Supplementary data:

**Table 1S (supplementary)-Change in anthropometric parameters after dietary intervention.**

| Anthropometric parameters | N | Mean± SD | | | P value  (Wilcoxan-signed rank test) |
| --- | --- | --- | --- | --- | --- |
|  |  | Baseline | Followup | Change |  |
| Weight | 34 | 15.53±15.02 | 17.87±14.71 | 2.33± 1.20 | **0.0001** |
| Weight for age | 27 | -1.74±1.13 | -1.33±1.10 | 0.40±.74 | **0.01** |
| Height | 34 | 92.64±36.94 | 97.55±34.14 | 4.90± 4.71 | **0.0001** |
| Height for age | 34 | -1.47±0.98 | -1.28±1.11 | 0.19±0.64 | 0.11 |
| Weight for height | 23 | -1.43±1.19 | -1.04±0.97 | 0.39± 1.23 | 0.10 |


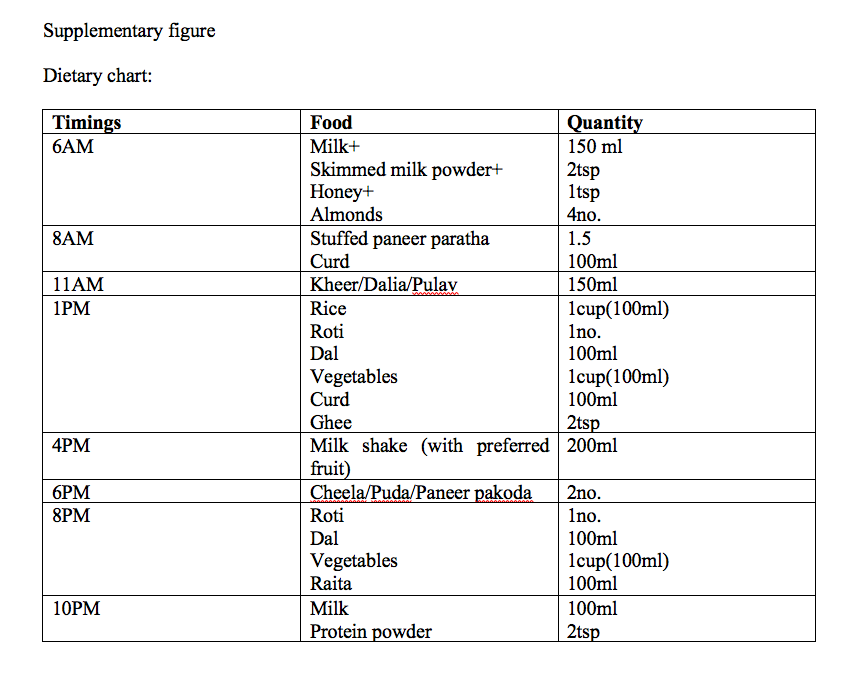


Figure 1S (supplementary) – Typical diet chart provided to the patient/care giver
